# Organic Selenium Ameliorates *Staphylococcus aureus*-Induced Mastitis in Rats by Inhibiting the Activation of NF-κB and MAPK Signaling Pathways

**DOI:** 10.3389/fvets.2020.00443

**Published:** 2020-07-30

**Authors:** Kangjun Liu, Tao Ding, Li Fang, Luying Cui, Jun Li, Xia Meng, Guoqiang Zhu, Chen Qian, Heng Wang, Jianji Li

**Affiliations:** ^1^College of Veterinary Medicine, Yangzhou University, Yangzhou, China; ^2^Jiangsu Co-innovation Center for Prevention and Control of Important Animal Infectious Diseases and Zoonoses, Yangzhou, China

**Keywords:** organic selenium, selenohomolanthionine, mastitis, *Staphylococcus aureus*, NF-κB, MAPK

## Abstract

Mastitis is an economically important disease in dairy cows, which is often caused by *Staphylococcus aureus* (*S. aureus*). Selenium is an indispensable element for physiological function and contributes to reduce injury of the mammary glands in mastitis. However, adequate sources of selenium have always been an important consideration for livestock. Therefore, the study aimed to explore the protective effect and mechanism of Selenohomolanthionine (SeHLan) on mastitis induced by *S. aureus*. The *S. aureus*-induced rat model was established and three doses (0.2, 2, 20 μg/kg body weight/day) of dietary OS were supplemented. The bacterial load, histopathology, and myeloperoxidase (MPO) of the mammary glands were performed and determined. Cytokines, including interleukin (IL)-1β, TNF-α, and IL-6, were detected using qRT-PCR. The key proteins of NF-κB and MAPK signaling pathways were analyzed by Western blot. The results revealed that OS supplementation could reduce the recruitment of neutrophils and macrophages in mammary tissues, but did not decrease *S. aureus* load in the tissues. The overexpression levels of IL-1β, TNF-α, and IL-6 induced by *S. aureus* were inhibited after OS treatment. Furthermore, the increased phosphorylation of NF-κB and MAPKs proteins were also suppressed. The results suggest that dietary supplementation with adequate OS during pregnancy contributes to protect the mammary glands from injury caused by *S. aureus* and alleviate the inflammatory response.

## Introduction

Mastitis, inflammation of the mammary glands, remains a significant health problem in both human beings and animals (1, 2). In cattle, mastitis is recognized as one of the most costly inflammatory diseases, which compromises the cow welfare and food safety (3). Bacterial infection is the main etiology in bovine mastitis and a wide variety of pathogens have been reported to cause the disease (4). *S. aureus* is one of the most prevailing pathogenic bacteria, which causes clinical and subclinical mastitis in dairy herds (5, 6). It was characterized by chronic, recurrent and lower cure rate in mastitis induced by *S. aureus* (7, 8). Although vaccines have been used to prevent mastitis caused by *S. aureus*, bovine mastitis mainly depends on antibiotic treatment. However, neither is effective in preventing *S. aureus* mastitis (9). Furthermore, ineffective treatment can also induce antimicrobial resistance in bacteria and drug residue in the products (10). So, it is necessary to develop alternative or supportive approaches to reduce the antibiotic usage in dairy cows.

There were extensive investigations in the defense mechanisms of the mammary glands. The successful triggering of pattern recognition receptors (PRRs) in both immune and non-immune cells by their ligands activates the downstream of nuclear transcription factor-kappa B (NF-κB) and mitogen-activated protein kinase (MAPK) signaling pathways, which initiate the inflammatory cascade by promoting the production of cytokines (11). Cytokines facilitate the recruitment of leukocytes at the infectious sites, which play an essential role in the early stage of *S. aureus* infection (12). Neutrophils are the main defensive cells during the early stage of the infection (13). The process of mastitis can be influenced not only by bacterial virulence factors but also by host-related factors, including the nutrition conditions from involution to lactation (12).

Selenium is necessary to maintain excellent reproductive performances in dairy cattle due to its beneficial biochemical and pharmacological properties (14, 15). And Se supplementation showed protective effects on the mammary gland health, which decreased the somatic cells count and incidence rate of the mastitis (16). SeHLan is a new selenoamino acid discovered in selenized Japanese pungent radish (17). Organic selenium supplementations, also including selenomethionine (SeMet), are mainly derived from Se-rich yeast *Saccharomyces cerevisiae*. However, SeHLan is obtained from *Candida utilis* (18). The metabolic pathway of SeHLan is simpler than SeMet. There are three metabolic pathways of SeMet, including trans-selenation for selenoprotein synthesis, replacing methionine for peptide synthesis, and methylation to form selenide or dimethylselenide. In contrast, SeHLan is only involved in the trans-selenation pathway, so SeHLan is affecting the metabolic pathway of methionine less and is more effective for production of selenoprotein (19–21). A previous study reported that SeHLan showed different tissue accumulation from SeMet in rats. SeMet was more easily distributed in the pancreas and caused pancreas damage, whereas SeHLan was preferably distributed in kidney and caused kidney damage. But the kidney damage can be avoided when SeHLan was administered at a lower dose than 1.0 mg Se/Kg body weight (19). It has been reported that SeHLan increased the survival rate of septic mice from 0.1 to 0.5 within 72 h after intraperitoneal injection of LPS, which was more effective than SeMet and selenite (22). So SeHLan is expected to be a potential Se source for livestock. As a new Se source, SeHLan has been given in dairy cows. However, the protective effects of SeHLan on the mammary glands during mastitis are unclear. So, the study was conducted to investigate what to the functions SeHLan played during mastitis induced by *S. aureus* and explore the potential mechanism using a rat mastitis model.

## Materials and Methods

### Materials

Organic selenium (purity ≥ 98%) was supplied by ABNA Trading Co., Ltd. (Shanghai, China). The organic selenium was derived from *Candida utilis* with analysis of 4,000 mg/kg of total Se and the main form of organic selenium is SeHLan (not <75%). The primary antibodies of IκB-α (#4812), p-IκB-α (#2859), p65 (#8242), p-p65 (#3033), JNK (#9258), p-JNK (#4668), ERK (#4695), p-ERK (#4370), p38 (#8690), p-p38 (#4511), β-actin (#4970) were provided by Cell Signaling Technology company (Boston, MA, USA). The myeloperoxidase assay kits were obtained from Nanjing Jiancheng Technology Co., Ltd. (Nanjing, China).

### Animals and Experimental Groups

Thirty pregnant Wistar rats of 8–10 weeks old (weight: 180–220 g) were obtained from the Laboratory Animal Center of Yangzhou University (Yangzhou, China). They were raised in standard plastic cages under a 12 h light/dark cycle. The room temperature was kept at 23 ± 2°C with a relative humidity of 55 ± 5%. A basal diet and distilled water were provided *ad libitum*. The rats were randomly divided into 5 groups (*n* = 6) as following: (a) control group, (b) *S. aureus* group, (c–e) *S. aureus* + OS-supplemented groups (0.2, 2, 20 μg/kg body weight/day, dissolved in distilled water). From the 1st day of pregnancy, rats in the groups(c-e) were administrated with OS orally until the end of the experiment. On the 4th day after parturition, the rats were anesthetized with isoflurane. L4 and R4 mammary glands respectively in the groups (b–e) were infused with 100 μL *S. aureus* suspension (2 × 10^7^ CFU/ mL) via the teat duct. The rats in the control group were infused with equal volumes of saline. Finally, all the rats were anesthetized with isoflurane at 12 h post infection. Part of the mammary tissue samples were collected aseptically for bacterial load. Some mammary tissue samples were prepared for histological studies. Other mammary tissues were immediately stored at −80°C.

### Determination of the Bacterial Load in the Mammary Glands

The *S. aureus* load in the mammary glands were detected as described previously (23). Briefly, the mammary tissues were homogenized in 1 mL phosphate buffered saline using a tissue grinder. The gland homogenates with 10-fold serial dilution method were plated on LB agar plates, incubated at 37°C. Then *S. aureus* colonies were counted.

### Histopathological Analysis

The mammary tissues were trimmed and fixed in 10% formalin. Then the tissues were embedded with paraffin wax and dehydrated in the graded alcohols. The sections were obtained and stained with hematoxylin and eosin (H&E). Histopathological changes were discovered and pictures were taken under a light microscope (Olympus, Japan).

### Determination of Myeloperoxidase Activity

Myeloperoxidase (MPO) activity in the mammary tissues were detected using the commercial kits according to the manufacturer's instructions. Briefly, the mammary gland tissues were homogenized with the extraction buffer (1:19, *w/v*). Then the homogenate was mixed with reaction buffer (9:1, *v/v*), and cultured at 37°C for 15 min. The enzymatic activity was calculated according to the absorbance at 460 nm.

### Quantitative Real-Time Polymerase Chain Reaction (qRT-PCR) Analysis

Trizol reagent was used to extract total RNA from the mammary tissues (Vazyme Biotech Co., Ltd, China). The concentration and purity of RNA were measured using NanoDrop 2000 spectrophotometer (Thermo Scientific, USA) as described previously (24). The RNA samples of A260/280 ratio ranging from 1.90 to 2.0 were selected for cDNA synthesis using HiScript® QRT SuperMix (Vazyme Biotech Co., Ltd, China). The specific primers used in this study were listed in Table 1, according to literature reports (25). The qRT-PCR was conducted using the CFX Connect^TM^ Real-Time System (Bio-Rad Instruments, Hercules, CA, USA) and ChanQ^TM^ SYBR® Qpcr Master Mix in a 20 μL reaction systems according to the recommended conditions (Vazyme Biotech Co., Ltd, China). The results were analyzed using the 2^−ΔΔ*Ct*^ method as previous description (26).

**Table 1:** The sequences of qRT-PCR primers.

**Genes**	**Forward (5'-3')**	**Reverse (5'-3')**	**GenBank accession no**.
TNF-α	GTAGCCCACGTCGTAGCAA	AAGTGGCAAATCGGCTGAC	NM_012675.3
IL-1β	GCAATGGTCGGGACATAGTT	GACTTGGCAGAGGACAAAGG	NM_031512.2
IL-6	CACAAGTCCGGAGAGGAGAC	ACAGTGCATCATCGCTGTTC	NM_012589.2
β-actin	TCTATCCTGGCCTCACTGTC	AACGCAGCTCAGTAACAGTCC	NM_031144.3

### Western Blot Analysis

The mammary gland tissues were homogenized with the RIPA lysis buffer containing protease and phosphatase inhibitors, and then centrifuged at 12,000 r/min for 15 min at 4°C. The protein concentration was determined using a BCA protein assay kit (Beyotime Biotechnology Co. Ltd, Shanghai, China). Equal amounts of protein were separated in 10% SDS-polyacrylamide gels and transferred to polyvinylidene difluoride membrane (Millipore, Germany). Subsequently, Tris-buffered saline containing 0.05% Tween 20 (TBST) and 5% skimmed milk were used to block the membranes at room temperature for 1 h. The membranes were incubated with primary antibodies against p65, p-p65, IκB-α, p-IκB-α, JNK, p-JNK, ERK, p-ERK, p38, p-p38, and β-actin (all at 1:1000 dilution in 5%BSA) at 4°C overnight, followed by incubation with secondary antibodies, conjugated with horseradish peroxidase, at room temperature for 1 h. Finally, the blots were visualized using enhanced chemiluminescence (ECL) assay according to the manufacturer's instructions. Bands were scanned and analyzed using Image J. β-actin antibody was used to prove equal loading of blots.

### Statistical Analysis

Data were expressed as the mean ± standard error of the mean (SEM) from at least three independent experiments. The obtained data were analyzed by SPSS Statistics 22.0 software (IBM, USA). Statistical significance was analyzed using one-way factorial analysis of variance (ANOVA) and evaluated using Tukey's multiple-comparisons test. *P* < 0.05 was considered statistically significant.

## Results

### Effects of OS on Bacterial Load of the Mammary Glands

**Figure 1:**
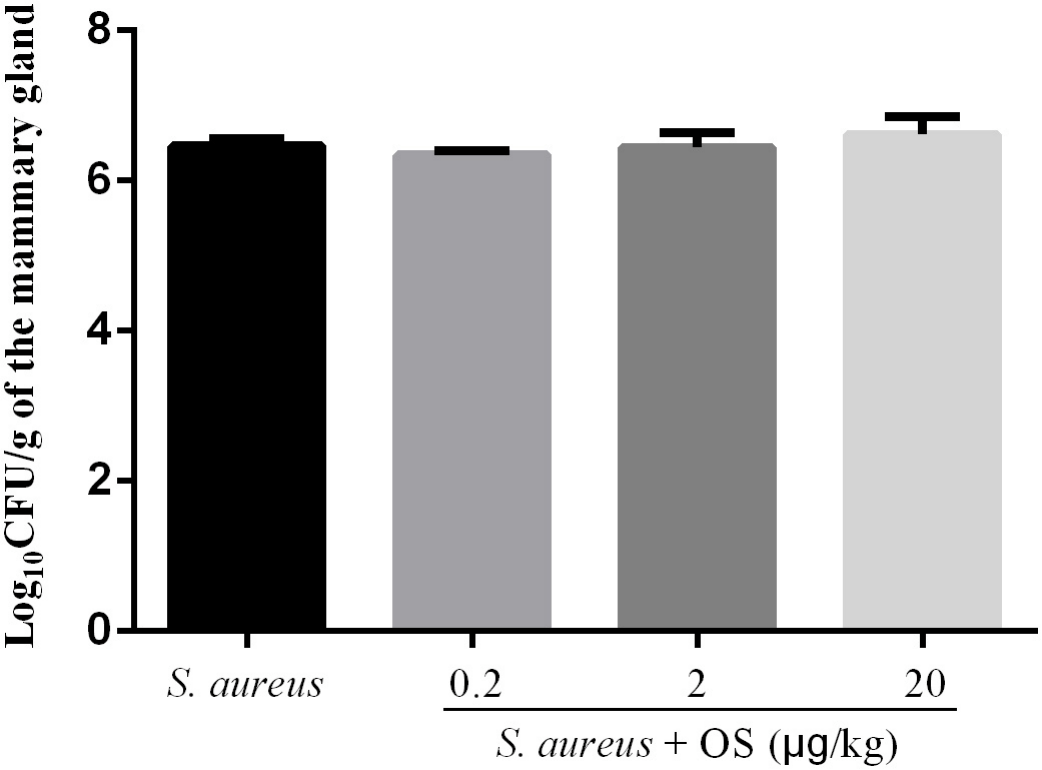
*S. aureus* load in the mammary glands. Values are presented as the mean Log_10_CFU/g of the mammary gland ± SEM (*n* = 5).

In order to verify whether OS contributes to the mammary gland against *S. aureus* infection, *S. aureus* load of the mammary glands were detected at 12 h after infection. No bacterial colonization was observed in the control group. Compared with the *S. aureus* group, low-dose OS supplementation reduced *S. aureus* load in the mammary glands slightly, but there was no significant difference. In middle- and high-dose OS-supplemented groups, there were no significant decrease in *S. aureus* load of the mammary glands compared to *S. aureus* group (Figure 1).

### Histopathological Changes

**Figure 2:**
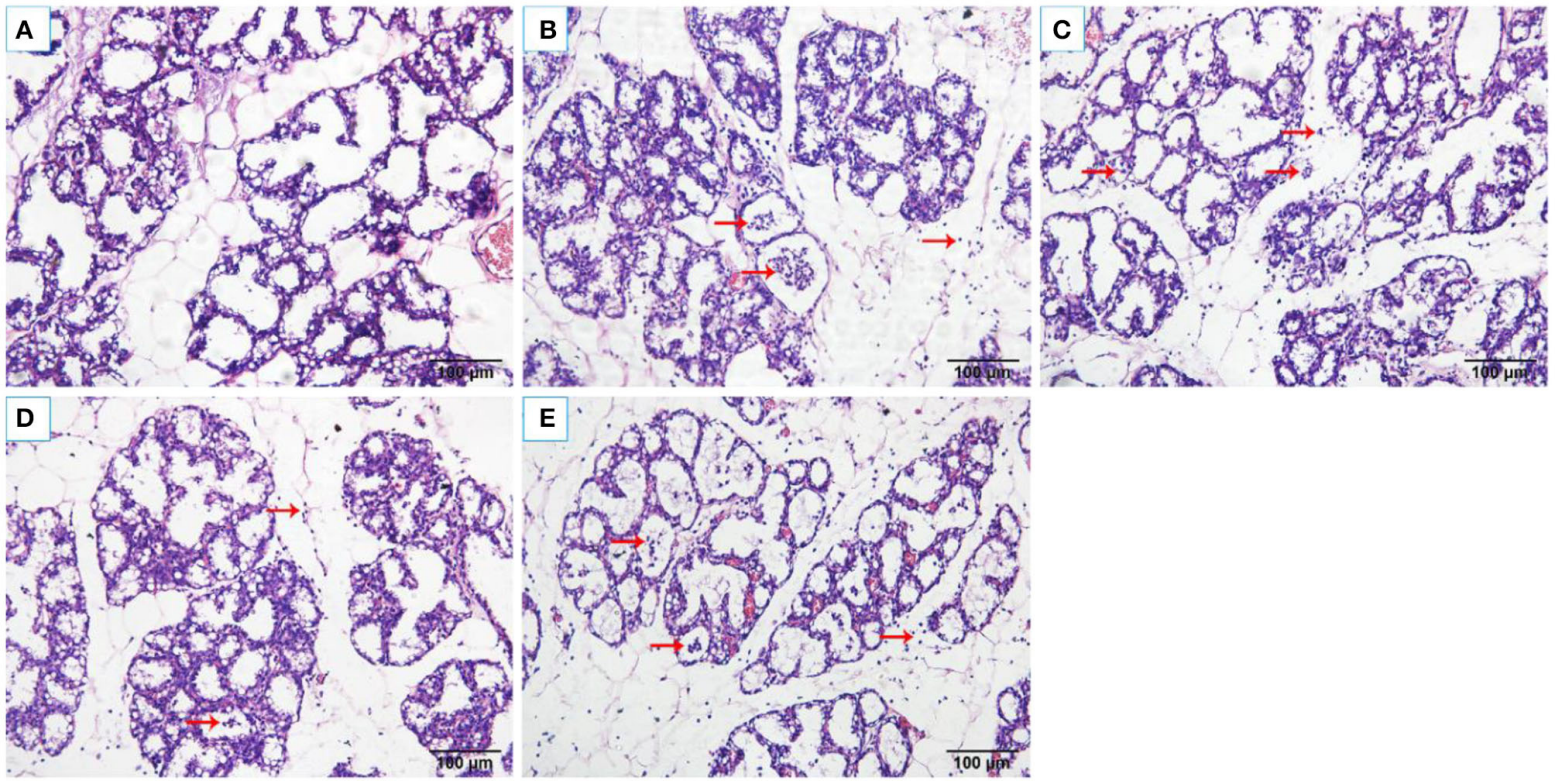
Histological changes of the rat mammary tissues (H&E 200×). **(A)** control group. **(B)** *S. aureus* group. **(C–E)** *S. aureus* + OS-supplemented groups (0.2, 2, 20 μg/kg body weight/day, respectively). The red arrows indicate the inflammatory cells infiltration and some debris of the mammary epithelial cells in mammary tissues.

The histopathological changes of the mammary tissues were evaluated as shown in Figure 2. There were no histopathological changes in the control group (Figure 2A). However, obvious inflammatory changes were observed in mammary tissues of *S. aureus* group, which were characterized by neutrophils and macrophages infiltrating in the alveoli lumens, ducts, perivascular, and connective tissues (Figure 2B). While, OS reduced the infiltration of immune cells and alleviated damage of the mammary glands (Figures 2C–E).

### MPO Activity

**Figure 3:**
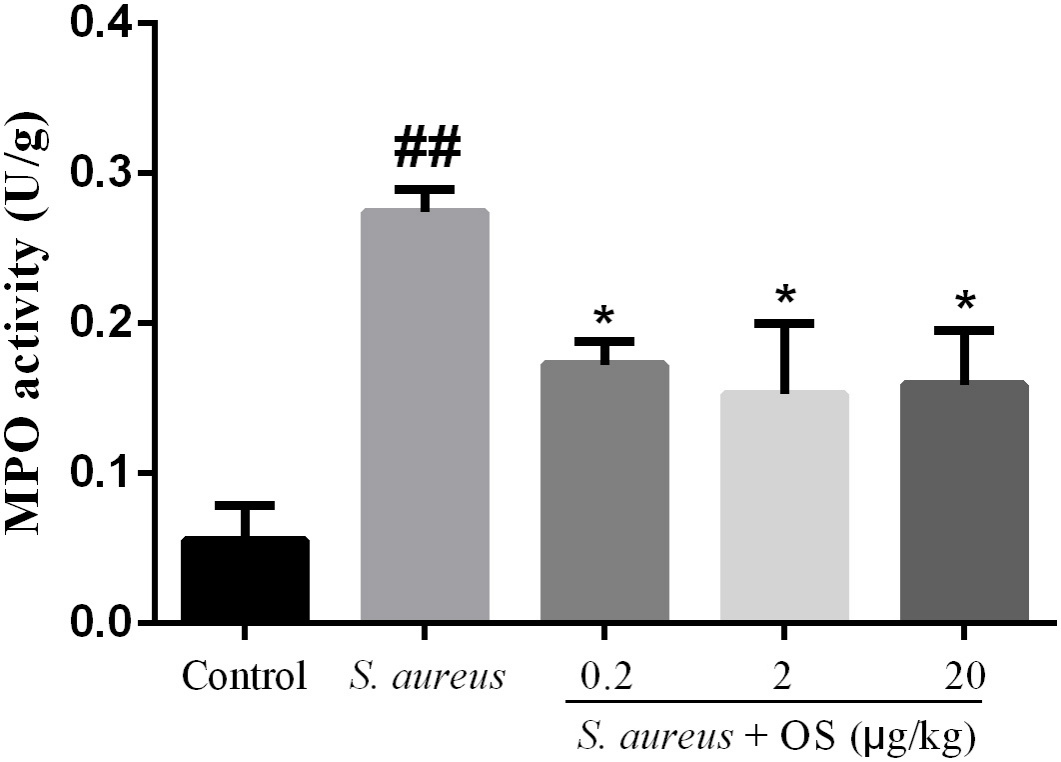
MPO activity in mammary tissues. Data are mean ± SEM (*n* = 5), ^##^*p* < 0.01 vs. control. *p < 0.05 vs. *S. aureus*.

MPO activity was used as a biomarker for the infiltration of neutrophils and macrophages, which plays important roles in evaluating the development of mastitis (27). The activity of MPO was detected in mammary tissues (Figure 3). Compared with the control group, the activity of MPO increased significantly after *S. aureus* infection (*p* < 0.01). OS supplementation effectively depressed the activity of MPO induced by *S. aureus* (*p* < 0.05). There was no significant difference between different OS-supplemented groups, although 2 μg/kg OS treatment decreased the most in the MPO activity.

### Inflammatory Cytokines mRNA Expression Analysis

**Figure 4:**
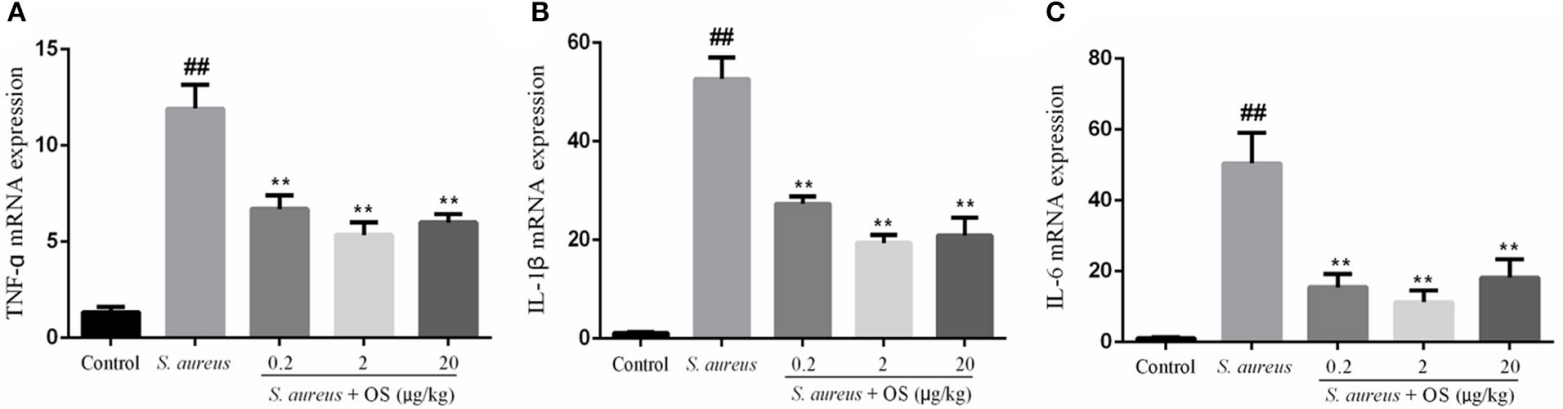
The effects of OS on the mRNA expression levels of TNF-α **(A)**, IL-1β **(B)**, and IL-6 **(C)**. Data are mean ± SEM, ^##^*p* < 0.01 vs. control. **p < 0.01 vs. *S. aureus*.

The effects of OS on the mRNA expression levels of TNF-α, IL-1β, and IL-6 induced by *S. aureus* were measured by qRT-PCR. As shown in Figure 4, the mRNA expression levels of TNF-α, IL-1β, and IL-6 in the *S. aureus* group increased significantly, relative to the control group (*p* < 0.01). Compared to the *S. aureus* group, OS significantly inhibited the mRNA expression levels of TNF-α, IL-1β, and IL-6 after *S. aureus* infection (*p* < 0.01). Similarly, the mRNA expression levels decreased most in the 2 μg/kg OS treatment group, but there was no significant difference relative to the other two OS-supplemented groups.

### OS Inhibits NF-κB Signaling Pathway Activation

**Figure 5:**
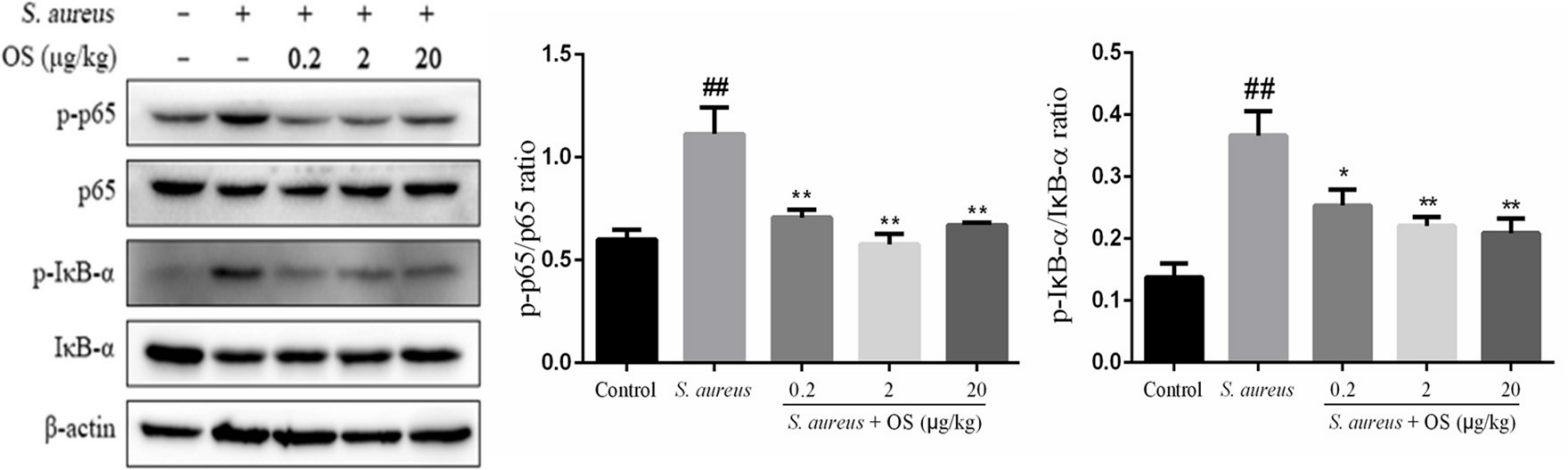
Effects of OS on *S. aureus*-induced NF-κB signaling pathway in the mammary glands. Western blot was used to detected the phosphorylation levels of p65 and IκB-α proteins. Data are represented as the mean ± SEM (*n* = 3). ^##^*p* < 0.01 vs. control. *p < 0.05 vs. *S. aureus*. **p < 0.01 vs. *S. aureus*.

To further determine the anti-inflammatory mechanism of OS, we evaluated the effects of OS on the phosphorylation levels of p65 and IκB-α proteins with western blot analysis. Phosphorylation of p65 and IκB-α proteins increased markedly after *S. aureus* infection, compared with the control group (*p* < 0.01). The phosphorylation of p65 protein was inhibited by OS supplementation with different concentrations (*p* < 0.01) when the mammary glands were infected by *S. aureus* (Figure 5). The middle-dose OS decreased the most in the phosphorylation of p65 protein, but no significant difference was observed compared to the low- and high-dose OS treatment. The phosphorylation level of IκB-α protein decreased significantly with the increase of OS concentration (*p* < 0.05, *p* < 0.01), compared with the *S. aureus* group. Therefore, OS could regulate the inflammatory response to some extent by inhibiting NF-κB signaling pathway activation.

### OS Inhibits MAPK Signaling Pathway Activation

**Figure 6:**
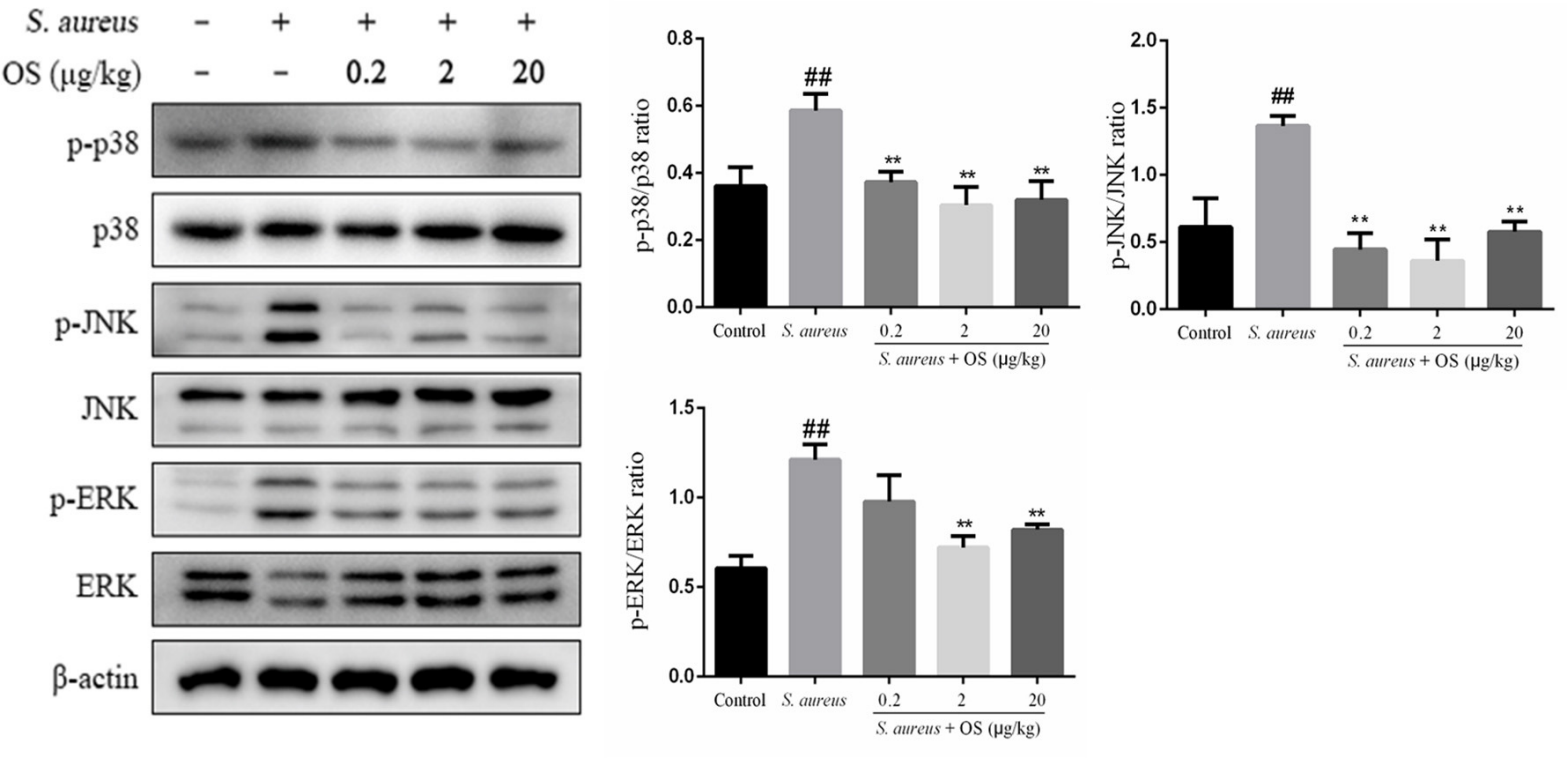
The effects of OS on MAPKs signaling pathway in the mammary glands. Western blot was used to assay the phosphorylation levels of p38, JNK, and ERK proteins. Data are mean ± SEM (*n* ≥ 3). ^##^*p* < 0.01 vs. control. **p < 0.01 vs. *S. aureus*.

In this study, the phosphorylation of p38, JNK, and ERK proteins were significantly upregulated after *S. aureus* infection, compared to the control group (*p* < 0.01). However, OS could inhibit *S. aureus*-induced phosphorylation of p38 and JNK proteins in different OS-supplemented groups (*p* < 0.01). And the phosphorylation of ERK protein was suppressed in middle- and high-dose OS-supplemented groups (*p* < 0.01) (Figure 6).

## Discussion

Mastitis is a common disease in dairy cattle negatively affecting economic return and animal health, which seriously hampers the development of dairy industries (28). *S. aureus* is one of the main pathogens involved in bovine intramammary infection (4). The infection not only affects the milk quality, but also poses a huge threat to food safety due to bacterial toxins and antibiotic residues (29, 30). Selenium was widely applied in dietary supplements for its antioxidative and anti-inflammatory effects (24, 31). In this study, the anti-inflammatory functions of OS were evaluated using a rat mastitis model induced by *S. aureus*.

It is essential to eliminate the pathogens to control the infection. It has been found that the bactericidal activity could be improved in cow milk whey with Se supplementation (32). It is unclear whether Se can reduce the bacterial load of the mammary glands after *S. aureus* infection. In this study, OS supplementation has no significant effect on reducing *S. aureus* load in the mammary glands. Histopathological examination showed that a large number of neutrophils and macrophages infiltrated mammary tissues after *S. aureus* infection, which were similar to previous studies (25, 33). But OS supplementation can reduce the infiltration of inflammatory cells and alleviate the tissue damage. The MPO activity was a typical marker of inflammatory cell infiltration (34). In line with histopathological observations, MPO activity increased significantly after *S. aureus* infection, but the increased MPO activity was suppressed in the OS supplemented groups. Therefore, OS supplementation alleviated the inflammatory response of mastitis induced by *S. aureus*, which may support the prevention and treatment of mastitis.

Inflammatory cytokines are essential for the initiation and development of mastitis (12). A previous study demonstrated that *S. aureus* was able to cause inflammatory responses by inducing the production of TNF-α, IL-1β, and IL-6 in the mammary glands of mice (24). In this study, the increased gene expression of TNF-α, IL-1β, and IL-6 were observed in the mammary glands of postpartum rats infected with *S. aureus*. TNF-α plays a key role in the initiation and orchestration of inflammation and immunity (32). In the earliest stage of inflammation, IL-1β acts as a trigger for a subsequent cascade of inflammatory cytokines (35). IL-6 increases quickly in acute inflammation, which is often associated with injury, infections, and other stress (36). The adequate production of cytokines is very important for immune response and host defense. However, persistent production of the inflammatory-related cytokines could result in swelling and rupture of the mammary cells, eventually causing tissue damage (37). The anti-inflammatory effects of Se compounds have been reported. Vunta et al. (38) reported that sodium selenite significantly decreased LPS-induced expression of TNF-α in bone marrow-derived macrophages. Ma et al. (24) found that Se-(methyl) selenocysteine hydrochloride had anti-inflammatory effects in *S. aureus*-induced mice mastitis via decreasing the gene expression levels of TNF-α, IL-1β, and increasing the gene expression level of IL-10. In our results, the overexpression of TNF-α, IL-1β, and IL-6 induced by *S. aureus* in the mammary glands of postpartum rats were downregulated after OS treatment across the given dose range. The results indicate that OS could alleviate the inflammatory response and the tissue damage by inhibiting the gene transcription of pro-inflammatory cytokines.

NF-κB is critical for the transcription of pro-inflammatory genes, which play a vital role in the amplification of inflammation (39, 40). The activation of NF-κB typically involves the degradation of IκB-α, followed by nuclear translocation of NF-κB p65 subunit (41). NF-κB dimers remain inactive by IκB proteins in the cytoplasm. When inflammation occurs, the IκB kinase triggers IκB-α ubiquitination and proteasome degradation via phosphorylation, which allows NF-κB p65 to accumulate in the nucleus to elicit the production of cytokines, such as TNF-α, IL-1β, and IL-6 (42). Previous studies have shown that *S. aureus* infection can increase the phosphorylation of IκB-α and p65 proteins, and activate NF-κB signaling pathway in lung and uterine tissues (43, 44). In this study, the phosphorylation of IκB-α and p65 proteins in mammary tissues were increased significantly after *S. aureus* infection, which indicated that NF-κB was involved in the inflammatory response. It has been demonstrated that SeMet was able to alleviate LPS-induced inflammatory response through inhibiting NF-κB activation in the chicken trachea (45). Wang et al. (46) observed Na_2_SeO_3_ suppressed *S. aureus*-induced NF-κB activation in bovine mammary epithelial cells. In this study, the increased phosphorylation of IκB-α and p65 proteins in the rat mammary glands infected with *S. aureus* were suppressed after OS supplementation. Combining with the inhibition of OS on inflammatory cytokines mRNA expression, our results indicated that the NF-κB signaling pathway was negatively regulated by OS in *S. aureus*-induced mastitis, at least partly, downregulation of the inflammatory genes expression and ultimately reducing the inflammatory response.

MAPK signaling pathway also plays a critical role in regulation of inflammatory response and the MAPKs p38, ERK, and JNK are the best-studied major MAP kinases (47). Upon pathogen challenge, p38 acts as a pivotal kinase involved in controlling the production of cytokines, such as TNFα, IL-1β, and IL-6 (48). JNK is also associated with regulating the expression and activation of inflammatory mediators (49). It has been revealed that ERK facilitates the development of inflammation-associated cancer by regulating the expression of inflammatory cytokines (50). Previous studies reported that the increased phosphorylation of the p38, ERK, and JNK proteins were involved in regulating the inflammatory response caused by *S. aureus* in mice mastitis (26) and pneumonia (51). In this study, the phosphorylation of p38, ERK, and JNK proteins were upregulated after *S. aureus* infection, which were similar to previous studies. It has been suggested that Se can reduce the inflammatory response caused by infection through regulating the MAPK signaling pathway. Liu et al. (52) reported that Se alleviated the inflammation induced by LPS via p38 MAPK in chicken myocardial. In RAW264.7 macrophages, MAPKs (p38, ERK, and JNK) took part in the inhibitory effects of Na_2_SeO_3_ on increased gene expression of cytokines induced by *S. aureus* (53). In this study, the elevated phosphorylation of MAPKs proteins induced by *S. aureus* were reversed by OS, indicating the involvement of MAPKs in OS-modulated anti-inflammatory effects in rat mastitis.

In conclusion, OS supplementation can suppress MPO activity and reduce the infiltration of inflammatory cells, thus protect mammary tissues from damage caused by excessive inflammatory response. The anti-inflammatory effects of OS on *S. aureus*-induced mastitis may be related to the process of suppressing the expression of inflammatory cytokine genes via inhibiting the activation of NF-κB and MAPK signaling pathways. The findings suggest that OS supplementation could be used as a potential treatment for mastitis.

## Data Availability Statement

All datasets generated for this study are included in the article/supplementary material.

## Ethics Statement

This study was approved by the Institutional Animal Care and Use Committee of Yangzhou University (No: YZUDWSY2017-0029).

## Author Contributions

HW and JiL conceived and designed the study. KL, TD, LF, LC, and JuL performed the experiments. KL, LC, and XM analyzed the data. KL wrote the manuscript. HW, JiL, GZ, and CQ revised it critically for important content. All authors read and approved the final version of the manuscript.

## Conflict of Interest

The authors declare that the research was conducted in the absence of any commercial or financial relationships that could be construed as a potential conflict of interest.
